# Measuring synaptic transmission and plasticity with fEPSP recordings in behaving mice

**DOI:** 10.1016/j.xpro.2021.101115

**Published:** 2022-01-20

**Authors:** Leore R. Heim, Shiri Shoob, Lior de Marcas, Daniel Zarhin, Inna Slutsky

**Affiliations:** 1 Department of Physiology and Pharmacology, Sackler Faculty of Medicine, Tel Aviv University, 69978 Tel Aviv, Israel; 2 Sagol School of Neuroscience, Tel Aviv University, 69978 Tel Aviv, Israel

**Keywords:** Microscopy, Neuroscience, Behavior

## Abstract

Spontaneous spiking activity depends on intrinsic excitability and synaptic input. Historically, synaptic activity has been mostly studied *ex vivo*. Here, we describe a versatile and robust protocol to record field excitatory postsynaptic potentials (fEPSPs) in behaving rodents. The protocol allows estimating the input–output relationship of a specific pathway, short-term and long-term plasticity, and their modulation by pharmacological or pharmacogenetic interventions and behavioral states. However, experimenters must be aware of the protocol’s specificity and interpret results with care.

For complete details on the use and execution of this profile, please refer to Styr et al. (2019).

## Highlights

• A protocol for recording and manipulating fEPSPs in behaving rodents
• Surgical and experimental procedures with tips on reducing noise and variability
• Pathway-specific fEPSP recordings following behavioral and chemogenetic manipulations
• Integration of pathway-specific fEPSP recordings and somatic Ca^2+^ imaging

## Graphical abstract

**Figure:**
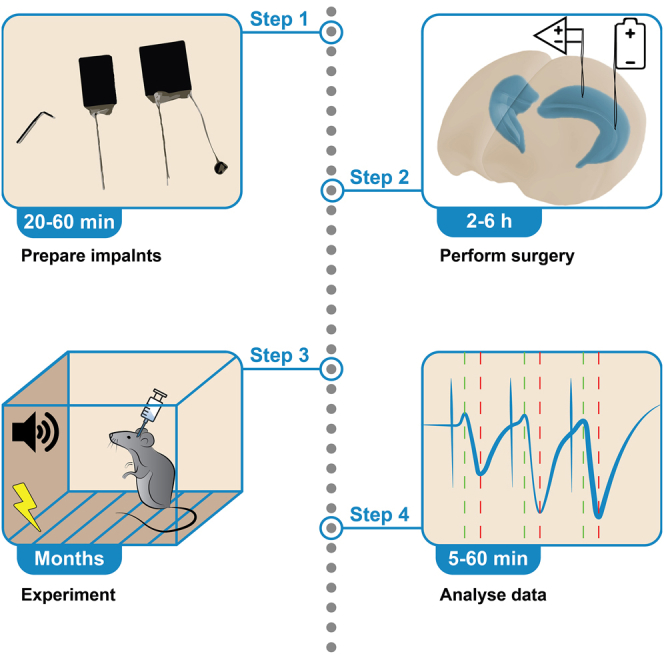

## Before you begin

In 2019, we performed a metabolic modeling analysis of epilepsy-associated transcriptome datasets which highlighted several metabolic genes as possible candidates for anti-epileptic therapy. To experimentally test their therapeutic potential *in vivo* and during different states of arousal, we adopted and improved existing protocols to record field excitatory postsynaptic potentials (fEPSP) of the Schaffer Collateral (SC) concurrently with pharmacological interventions. The result is a robust, low-cost, and extremely versatile method to study synaptic transmission in behaving rodents. Further, due to the small footprint of our custom fEPSP electrodes, this protocol can be easily combined with other methodologies such as tetrode microdrives (Voigts et al., 2020) or miniaturized microscopes (Aharoni and Hoogland, 2019) for simultaneous recordings of, e.g., evoked synaptic activity and calcium transients (Figure 11).

Alongside the aforementioned technical advantages, the scope of data that can be obtained from fEPSP recordings is exceptionally wide: In addition to subthreshold activity, suprathreshold activity can be probed when a population spike is recognized as part of the evoked activity waveform (Scherf et al., 2010). Further, both short-term plasticity (Figure 8) and long-term plasticity can be induced with the proper stimulation protocol. These measurements and modulations of synaptic activity can be used to test the effects of pharmacological (Styr et al., 2019), pharmacogenetic (Figure 9) and behavioral interventions (e.g., fear conditioning; Figure 10) since the method was attuned for behaving animals.

The protocol focuses on constructing the implants, performing the surgery, and running experiments. When possible, we exemplify the versatility of the method by proposing alternatives to the main experimental design. The protocol has been written with the intention to provide all information necessary to adopt the proposed method even by laboratories who never before performed electrophysiological experiments. For this purpose, we also provide a brief description of necessary and recommended laboratory facilities. The required skills from the readers are basic coding abilities in MATLAB for implementing our custom fEPSP analysis pipeline and basic soldering techniques for constructing the electrodes. The surgical procedure is depicted in full yet we highly recommend novice students to acquire hands-on training from experienced surgeons and consult with other resources dedicated to surgical techniques in rodents (Ferry et al., 2014).

### Animals

The protocol was designed for and tested on female and male mice aged 2–6 months, but can be easily adapted for rats and other small rodents. The animals may be commercially obtained (e.g., purchased from the Jackson Laboratory) or involve newly created transgenic lines.

***Note:*** All experiments must receive approval from the relevant institutional review board and be conducted in strict accordance with the guidelines for animal care provided by the National Institutes of Health (National Research Council Committee, 2011). All procedures performed here were approved by the Sackler Faculty of Medicine Ethics Committee (01-17-099).

### Electrophysiological setup

fEPSP signals between region A and region B are typically elicited by driving current between a pair of adjacent wires positioned in region A and recording the differential signal between a pair of adjacent wires positioned in region B. The fEPSP signal is then amplified and digitized before being stored on a computer. Thus, aside from the electrodes, fEPSP recordings require a stimulator, amplifier, digitizer, controller, and the appropriate data acquisition software. Numerous products exist that fulfill one or more of these tasks and that can be combined in many forms to assemble the electrophysiological setup. Table 1 depicts two such variations of electrophysiological setups. In setup variation no. 2, analog-to-digital conversion is done by a headstage connected directly to the electrodes. Accordingly, the cables connecting the animal to the electrophysiological setup transmit digital data which is less sensitive to electromagnetic interferences (troubleshooting 1). However, the headstage adds significant weight to the animal and thus may hinder its behavior, especially when used with small rodents.

***Note:***Table 1 does not list the cables used to deliver data between recording modalities and/or provide current to the stimulating electrodes. However, appropriate cables are typically included with the relevant instrument or can be easily built with the workshop tools listed in the Key resources table.

***Alternatives:*** Most components listed in Table 1 can be custom built using basic electronics. Such endeavors are typically very time consuming but provide the student responsible with invaluable engineering insights. From our experience, the quality of signals produced by custom built products (e.g. amplifiers) are comparable to those produced by commercially available alternative.

***Note:*** any PC that can carry MATLAB is sufficient for most acquisition software. However, we recommend recording the data on a solid-state drive (SSD) rather than a hard disk drive (HDD) as this may circumvent buffer issues and the accidental loss of data. In general, fEPSP recordings by themselves do not require much storage space (∼0.5 MB/min if acquired at a sampling frequency of 1000 Hz and saved as data type double). However, if fEPSP signals are to be combined with other recording modalities, we recommend dedicating ∼250 GB of SSD storage space for the acquisition and short-term storage of raw data.

**Table 1:** Two variations of electrophysiological setups

Setup no.	1	2
Amplifier	Model 1700 by A-M Systems	RHD16ch with bipolar inputs by Intan
Digitizer	Digidata 1440A by Molecular Devices	RHD16ch with bipolar inputs by Intan
Stimulator	DS3 by Digitimer	Model 2100 by A-M systems
Controller	Digidata 1440A by Molecular Devices	Arduino Uno microcontroller and RHD2000 controller by Intan
Software	WinWCP	Arduino IDC and Intan RHX software

Table 2 lists several typical stimulation protocols used to elicit the fEPSP signal during implantation and experimentation. These stimulation protocols must be configured in the electrophysiological setup before starting the surgery. For convenience, stimulation protocols written for winWCP and Arduino can be found in the accompanying GitHub repository (see resource availability).

**CRITICAL:** The absolute value of current used to elicit fEPSP signals varies between electrodes, electrophysiological setups, and brain regions. Given all three of these parameters are kept constant, we expect stimulus intensities to not vary between mice by more than 30%. Typical values for various synapses are between 0.04–0.12 mA (Figure 7) and should never exceed 0.5 mA when electrodes are positioned correctly. Calibrating the precise range of intensities for an individual mouse is addressed in steps 13, 19, and troubleshooting 2.

***Alternatives:*** Pulse duration for both I/O and STP recordings is typically 500 us. However, 100 us pulses at higher stimulus intensities can also be used.

***Note:*** Full characterization of I/O curves (e.g. Figure 7B) should include 5–10 stimulus intensities ranging from no fEPSP response up until saturation of the fEPSP response (i.e. no increase in amplitude in response to an increase in stimulus intensity).

***Note:*** STP recordings require that the amplitude of the first evoked response to the train of stimuli be noticeable yet below saturation (Figure 8A). If the experimental manipulation (e.g. candidate drug) is expected to significantly change synaptic transmission (i.e. the evoked response amplitude), it is advised to record baseline STP signals with two or more stimulus intensities so that after the manipulation there will be at least one intensity to which the amplitude of the first response remains within the aforementioned boundaries. In addition, 50 Hz as the stimuli frequency is commonly used to elicit synaptic facilitation in the SC, but other frequencies may be preferred for other pathways that display different frequency-dependent response.

**Table 2:** Stimulation protocols

Protocol	Description	No. of increasing stimulus intensities	No. of repetitions per intensity
Input-output (I/O)	1× square pulse @ 0.06 Hz	5–10 intensities	5–10 once every 15 s
Short-term plasticity (STP)	3× square pulses @ 50 Hz	1–3 intensities	3–8 once every 30 s

### Drug delivery system

Systemic delivery of pharmaceuticals is typically achieved by manually administering an intraperitoneal (i.p.) or subcutaneous (s.c.) injection with an insulin needle. Local delivery of pharmaceuticals to the ventricles (intracerebroventricular; i.c.v.) or brain parenchyma demands more precise control over the volume delivered (typically 100–1000 nL) and delivery rate (typically 50–200 nl/min). This requires a drug delivery system comprised of a pump (e.g., UMP3 by World Precision Instruments), a controller (MICRO2T by World Precision Instruments), a syringe (e.g., 701RN by Hamilton), and a needle (e.g., 7803-03 by Hamilton). In chronic experiments, the needle is directed with stereotaxic instruments towards the target region via a cannula secured to the animal’s skull during surgery (see step-by-step method details).

**Figure 1:**
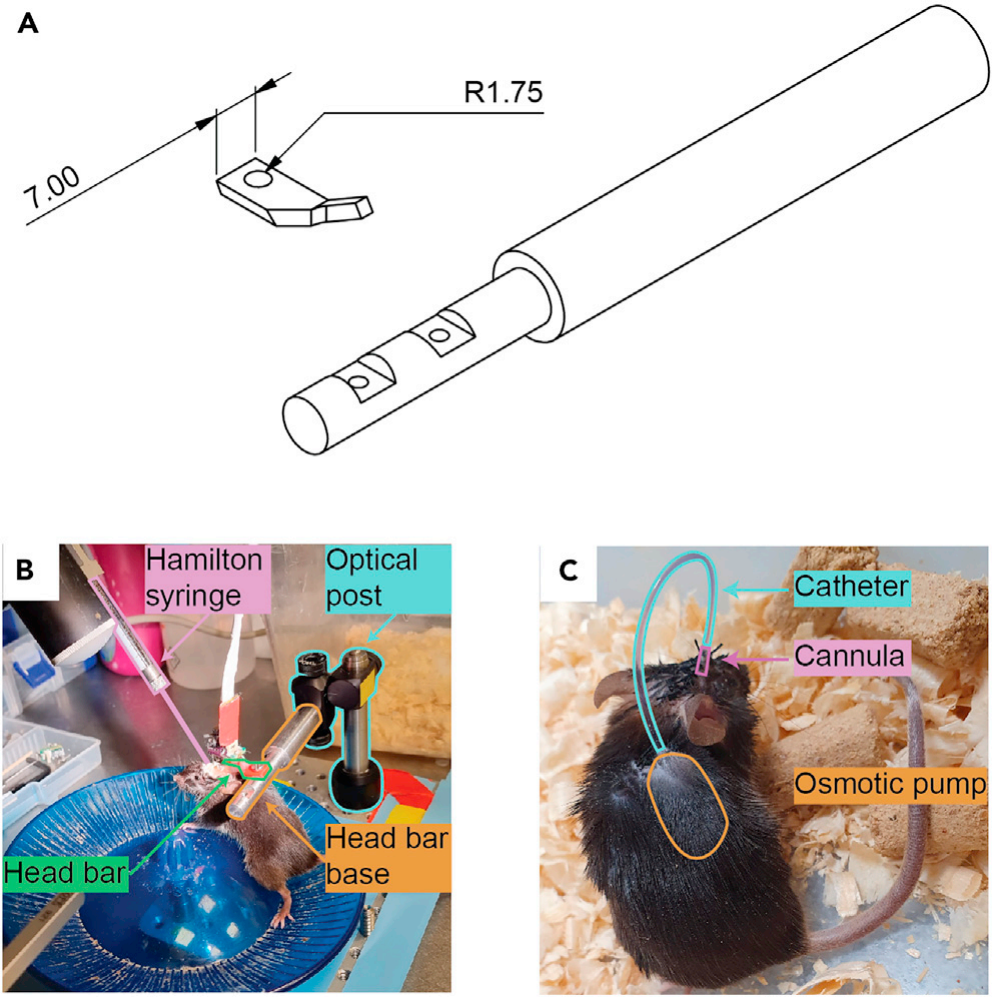
Head fixation apparatus (A) Schematics of custom components. CAD files are available in the accompanying repository (see resource availability). (B) A mouse connected to the head fixation apparatus during intracerebral injection. (C) An osmotic pump connected via catheter to a cannula implanted within the brain parenchyma.

Stereotactic injections require that the animal’s head be completely immobile. However, repetitive administration of anesthetics risks the animal and confounds the data. Thus, we developed a simple head fixation apparatus (Figure 1) based on several commercially available components alongside two custom made components that can be 3D printed or, preferably, machined from stainless steel (Key Resources Table).

***Alternatives:*** Long-term continuous delivery of pharmaceuticals can be achieved by implanting an osmotic pump subcutaneously (e.g. Alzet 1007D). To bypass the blood-brain barrier, the drug can be directed from the osmotic pump to an implanted cannula with a catheter (e.g. Alzet #0007760l; Figure 1C).

### Prepare for surgery

**Timing: 30–120 min**

**Figure 2:**
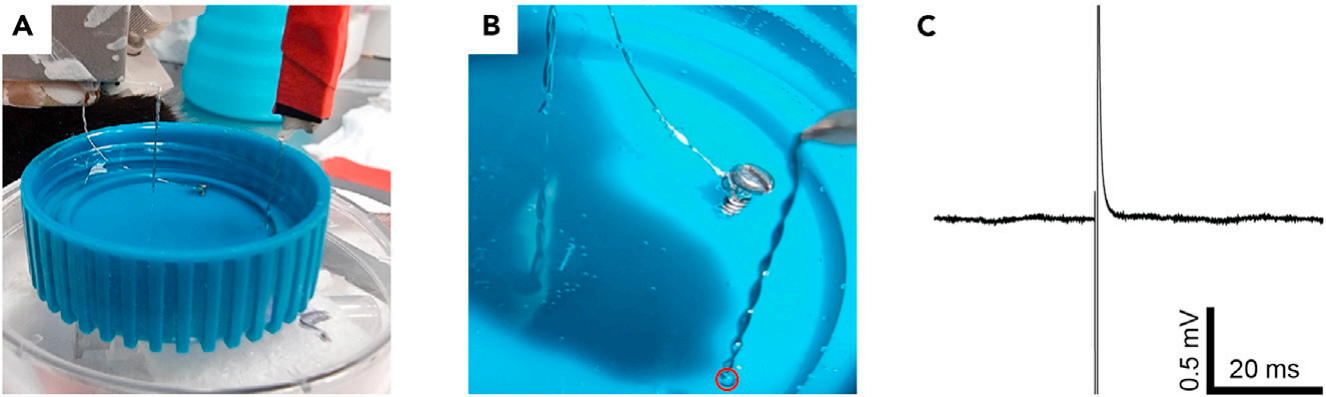
Testing the electrodes and electrophysiological setup before surgery (A) Immerse the electrodes in PBS. (B) Deliver 0.04–0.1 mA of current continuously for 2–5 s to the stimulating electrode and note air bubbles (red circle) form at the electrode tip due to electrolysis of water molecules. (C) Deliver a 0.5 ms square pulse of current at 0.04–0.1 mA and note that the recording electrode captures a detectable artifact.
  b. Record a continuous signal and inspect its quality in terms of root mean square (RMS) and electromagnetic interferences (see troubleshooting 1).
  c. Deliver constant current at 0.04–0.1 mA to the stimulating electrodes and observe under the microscope that air bubbles emerge from the wire tips (Figure 2B).
  d. Deliver a 0.5 ms square pulse of current at 0.04–0.1 mA to the stimulating electrodes and note that the recording electrode captures the stimulus artifact (Figure 2C).
  e. Test that the stimulating protocols are configured properly (Table 2).
  f. Sterilize the implants (electrodes, cannula/s, and ground screw) with 70% ethanol.

***Note:*** It is highly recommended to prepare and test at least one spare of each implant.

**Figure 3:**
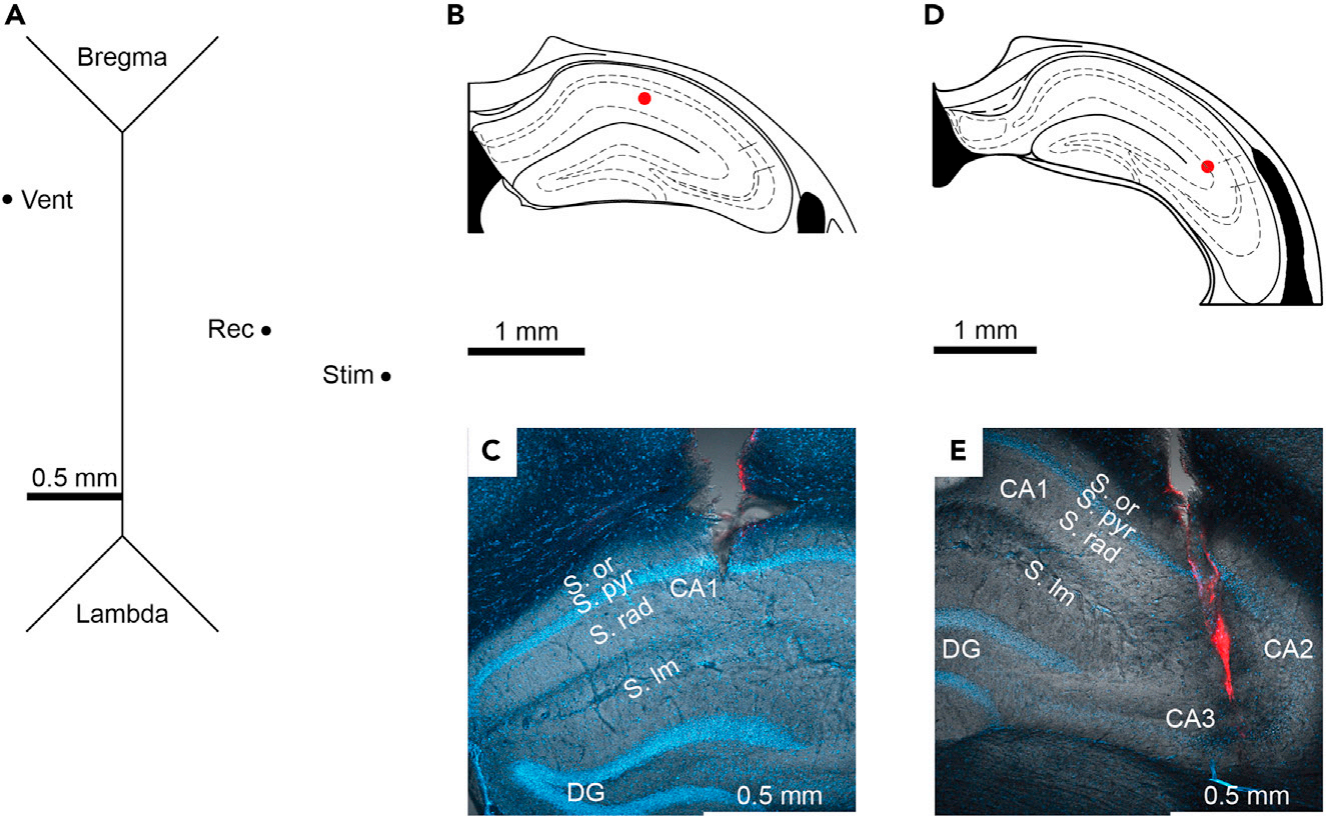
Implant coordinates (A) Schematic of a mouse skull with markings of craniotomies for a cannula in the left ventricle (Vent), a recording electrode in CA1 *stratum radiatum* (Rec), and a stimulating electrode in the SC (stim). The thick black line is positioned at the interaural line and represents a scale of 0.5 mm. (B) Schematic of a coronal slice of the right hippocampus ∼2 mm posterior to bregma demonstrating the target location for the recording electrode in *CA1 stratum radiatum* (red circle). Adapted from (Paxinos and Franklin, 2004). (C) Image of a coronal slice from a mouse implanted with a recording electrode in CA1 *stratum radiatum*. Before implantation, electrodes were dipped in a red labeling solution (Vybrant CM-Dil by Invitrogen) and the slice was stained with DAPI. S. or – *stratum oriens*; S. pyr – *stratum pyramidale*; S. rad – *stratum radiatum*; S. lm – *stratum lacunosum moleculare*; DG – dentate gyrus. (D) Schematic of a coronal slice of the right hippocampus ∼2.5 mm posterior to bregma demonstrating the target location for the stimulating electrode in the SC (red circle). Adapted from (Paxinos and Franklin, 2004). (E) Same as (C) but for the stimulating electrode in SR.
4. Prepare surgery station.
  a. Sterilize all relevant surgical tools.
  b. Check that the isoflurane syringe is properly filled.
  c. Prepare solutions (see materials and equipment).
  d. Check all electro-mechanical components (e.g., heating pad, micromotor drill, etc.).
  e. Check consumables (e.g., Gelfoam, applicators, etc.).

***Note:*** The key resources table in this study can be used as a checklist for all the necessary items during surgery.

1. Test the electrodes and electrophysiological setup.
  a. Connect the electrodes prepared in major step 1 to the electrophysiological system and place the electrode tips and ground screw in PBS. Make sure the recording and stimulating electrodes are in close proximity (∼5–20 mm; Figure 2A).

2. Prepare the animal.
  a. Weigh the animal and note his general wellbeing.
  b. Log all relevant information (genotype, age, etc.) in a dedicated notebook.
  c. Prepare in advance a home cage for the animal’s recovery after surgery.
3. Plan the craniotomies (Figure 3).
  a. Validate the coordinates and desired brain region/s using an atlas (Paxinos and Franklin, 2004).
  b. Draw a schematic of the skull and points of craniotomies.

5. Prepare yourself.
  a. Reserve in advance the surgery room with enough spare time.
  b. Ask an experienced surgeon to be present or available on the day of surgery.
  c. Make sure you are familiar with this protocol and any other relevant protocols or procedures.

***Note:*** Novice surgeons frequently have difficulties anticipating the duration of surgical procedures, especially procedures that involve electrophysiological recordings. For this reason, we highly recommend starting the surgery after eating and long (∼6 h) before the end of the day.

## Key resources table

**Table:** 

REAGENT or RESOURCE	SOURCE	IDENTIFIER
**Chemicals, peptides, and recombinant proteins**		

Isoflurane	Sigma-Aldrich	CAS: 26675-46-7
Buprenorphine	Sigma-Aldrich	CAS: 52485-79-7
Carprofen	Sigma-Aldrich	CAS: 53716-49-7
Ketamine hydrochloride	Sigma-Aldrich	CAS: 1867-66-9
Xylazine hydrochloride	Sigma-Aldrich	CAS: 23076-35-9
Hydrogen peroxide	Sigma-Aldrich	CAS: 7722-84-1
Povidone-iodine	Sigma-Aldrich	CAS: 25655-41-8
Ethanol 70%	Sigma-Aldrich	CAS: 109-56-8
Phosphate buffered saline (PBS)	Sigma-Aldrich	CAS: 7758-11-4
Adhesive luting cement	Parkell	C&B Metabond
Light curing glue	Pentron	Flow-it ALC A3
Hair removal cream	Veet	N/A
Duratears lubricating eye ointment	Alcon Laboratories	N/A
Gelfoam hemostatic sponge	Pfizer	Cat#9032301
Dental acrylic powder	Henry Schein	Cat#09-031
Dental acrylic fluid	Henry Schein	Cat#13-787

**Deposited data**		

CAD files of head fixation apparatus	This study	https://github.com/leoreh/slutsky_fepsp
Spreadsheet for atlas coordinates	This study	https://github.com/leoreh/slutsky_fepsp

**Experimental models: Organisms/strains**		

Mouse: C57BL/6J	The Jackson Laboratory	Strain#000664

**Software and algorithms**		

pCLAMP data analysis software	Molecular Devices	RRID: SCR_011323; https://www.moleculardevices.com/products/axon-patch-clamp-system/acquisition-and-analysis-software/pclamp-software-suite
Matlab programming platform	MathWorks	RRID: SCR_001622; https://www.mathworks.com/products/matlab.html
ImageJ image processing package	N/A	RRID: SCR_002285; https://imagej.nih.gov/ij/download.html
WinWCP	University of Strathclyde	https://github.com/johndempster/WinWCPXE/releases/tag/V5.6.6
Stimulating protocols in winWCP	This study	https://github.com/leoreh/slutsky_fepsp
Stimulating protocols in Arduino	This study	https://github.com/leoreh/slutsky_fepsp
fEPSP analysis package in MATLAB	This study	https://github.com/leoreh/slutsky_fepsp

**Other**		

Amplifier	A-M systems	Model 1700
Digitizer	Molecular Devices	Digidata 1440A
Stimulator	Digitimer	DS3
Micro syringe pump	World Precision Instruments	UMP3
Pump controller	World Precision Instruments	MICRO2T
Microliter syringe	Hamilton	Cat#701RN
Syringe needle	Hamilton	Cat#7803-03
Magnetic Stirrer	VELP scientifica	Cat#F203A0440
Rotary tool	Dremel	Cat#8300
Cutting wheel for rotary tool	Dremel	Cat#420
Side cutter	NWS	Cat#021F-79-ESD-115
Round nose pliers	NWS	Cat#021B-79-ESD-115
Microscope workshop	Olympus	Cat#SZ51
Soldering station	Weller	Cat#PU 81
Soldering flux	Indalloy	Cat#84003
Soldering wire	Multicore	Cat#362
LED curing light	COXO	Cat#DB-685
Helping hand	Allied electronics	Cat#JA-40
Air duster	Servisol	Cat#235
Sandpaper sheet 60 grit	N/A	N/A
Multimeter	Fluke	Cat#77-4
Plasticine	N/A	N/A
Adhesive tape	N/A	N/A
Slotted screwdriver 2 mm	Wera	Cat#2035
Machined screws	Palboreg Federal LTD	Size#M1x0.2; Standard#DIN 85
Coated stainless steel wire	A-M system	Cat#791400
3-pin female connector	Sullins Connector Solutions	Cat#S7036-ND
2-pin female connector	Sullins Connector Solutions	Cat#S7035-ND
Male connectors	Harwin Inc.	Cat#M20-9990545
Cannula tube	Component supply	Cat#HTX-23T-30
Filler rod	Component supply	Cat#HTX-28R-30
Isoflurane anesthesia vaporizer	Kent Scientific	SomnoSuite
DC temperature controller system	FHC	Cat#40-90-8D
Small animal stereotaxic instrument	Kopf Instruments	Cat#902
Ear bars	Kopf Instruments	Cat#1921
Mouse head holder	Kopf Instruments	Cat#923-B
Micromotor drill	Saeshin	Strong 90
Burr for micro drill	Fine Science Tools	Cat#19007-07
Fine scissors	Fine Science Tools	Cat#14060-11
Skin forceps	Fine Science Tools	Cat#11027-12
Fine forceps	Fine Science Tools	Cat#11231-30
Scalpel handle	Fine Science Tools	Cat#10003-12
Scalpel blades #15	Fine Science Tools	Cat#10015-00
Hemostat	Fine Science Tools	Cat#13008-12
Bulldog Serrefines	Fine Science Tools	Cat#18050-28
Cotton tip applicators	Cardinal health	Cat#C15055-006
Skin marker	Fine Science Tools	Cat#18000-30
Precision digital weight	American Weigh Scales	Cat#LB-3000
Insulin needles 31g	Novofine	N/A
Microscope surgery	Zeiss	Stemi 508
∗Optical construction post	Thorlabs	Cat#TR3T
∗Optical post	Thorlabs	Cat#TR75/M
∗Rotating clamp	Thorlabs	Cat#SWC/M
∗Post holder	Thorlabs	Cat#PH30/M
∗Running wheel	Bio-serv	Cat#K3251
∗Head bar	This study	https://github.com/leoreh/slutsky_fepsp
∗Head bar base	This study	https://github.com/leoreh/slutsky_fepsp

∗ Custom head fixation apparatus.

## Materials and equipment

**Table:** Solutions requiring preparation before surgery

Product	Final solution concentration	Recommended dose
Ketamine/Xylazine	Ketamine 10 mg/mL Xylazine 1.33 mg/mL	Ketamine 60 mg/kg Xylazine 8 mg/kg
Carprofen	0.5 mg/mL dissolved in PBS	0.125 mg/kg
Buprenorphine	0.01 mg/mL dissolved in PBS	0.05 mg/kg

***Note:*** We recommend that stock solutions are stored aliquoted in sealed vials at −20°C and used within one month. Alternatively, 5–10 mL of stock solution can be prepared and stored at 4°C if it is used within one week.

***Note:*** The table above lists the recommended dose and solution concentration of three drugs used in this protocol for anesthesia and analgesia. These should be prepared from the stock solution prior to surgery. Other drugs/ointments used throughout this protocol (e.g. eye ointment, povidone-iodine, etc.) are listed in the Key resources table and do not require any modification before use.

***Note:*** There are many pharmacological regimens for anesthetizing rodents and managing their pain. The regimen described here typically provides efficient anesthesia and analgesia while preserving a wide therapeutic window. Still, we recommend experimenters to consult with the veterinary service at their institute and make sure that the pharmacological treatment described here fits the specific needs of their animals and/or experiments.

## Step-by-step method details

### Building brain implants

**Timing: 20–60 min**

This step describes the construction of (1) a ground wire, (2) recording and stimulating electrodes, and (3) a cannula for local delivery of drugs. Stimulating and recording electrodes are identical except for the final cut (step 2.h). The implants described here can be modified to fit the specific experimental needs.

**Figure 4:**
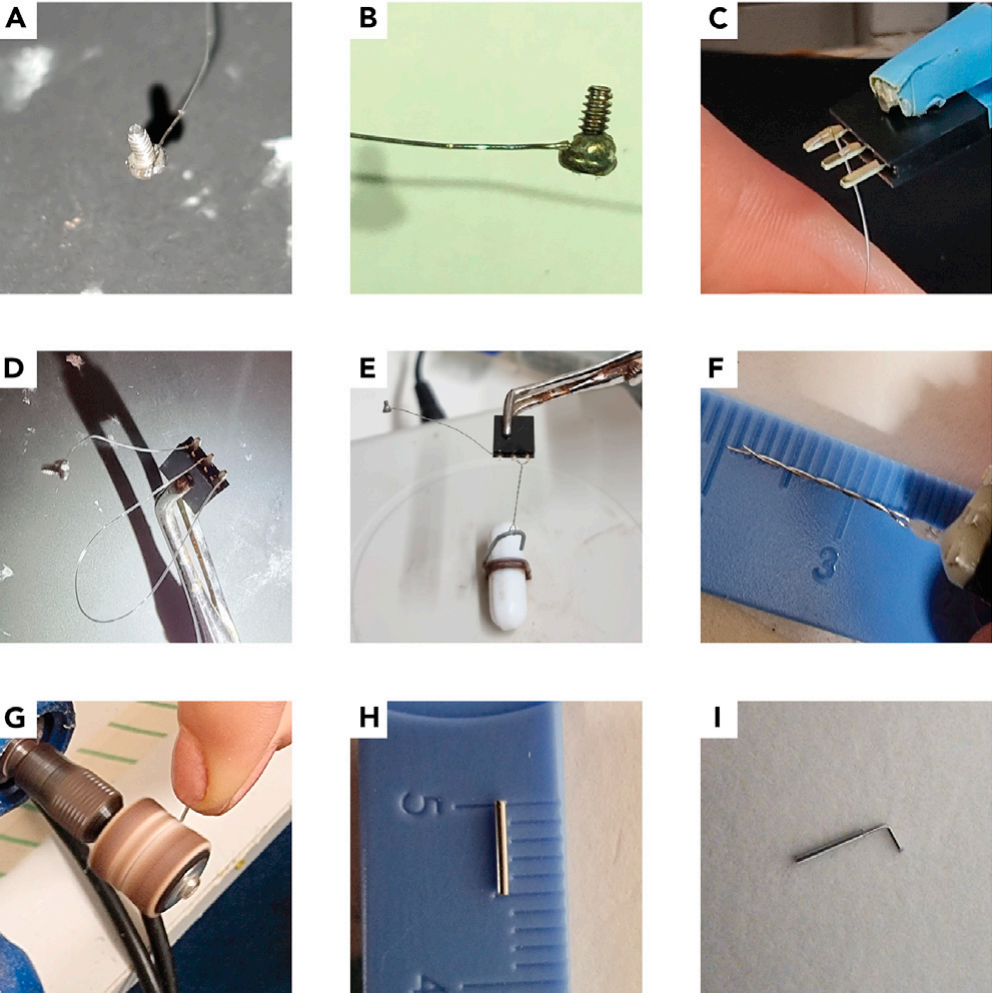
Building the implants (A) Solder the wire tip to the ground screw at a single point. (B) Wrap the wire around the screw base and solder them together. (C) Solder the other end of the ground wire to a connector pin. (D) Solder both ends of a wire to the remaining two pins of the connector. (E) Use a magnetic stirrer to twist the wire. (F) Cut the wire such that there is a 1mm height difference between the two tips. (G) File the sharp edges of the cannula tube with a rotary tool. (H) Measure and log the cannula length. (I) Prepare a filler rod and bend it before placing it within the cannula.
    iii. Wrap the wire around the head. Avoid circling the threads.
    iv. Solder the wrapped wire at a point other than the first.
    v. Make sure that both the drive and threads of the screw are clear from tin (Figure 4B).
    vi. Clean the screw from remnant soldering flux.
  d. Loop the free end of the wire around the tip of a thin tweezer. The loop should be big enough to surround a connector pin.
  e. Solder the looped end of the wire to a connector pin.
    i. Hold the connector with helping hands or a secured tweezers such that the pins are freely accessible.
    ii. Pass the connector pin through the wire loop (Figure 4C)
    iii. Apply soldering flux and solder the wire to the pin.
    iv. Cut the excess length of the pin with a cutter. This reduces the amount of glue that will be used in the next step.
  f. Cover the soldered pin with light curable glue and cure the glue. Use as little glue as possible to minimize mass and volume of the head construct.

***Note:*** Accurate soldering is more easily accomplished when only small amounts of soldering flux are applied with a fine (24–28G) needle.

1. Prepare a ground screw.
  a. Cut 4 cm of stainless-steel wire.
  b. Expose 3 mm from both sides of the wire.
    i. Secure the wire tightly between your fingernails or a hemostat.
    ii. Place the wire 3 mm within fine tweezers and apply a small amount of pressure.
    iii. Rapidly pull the tweezers such that the protective sheet is removed from the edge of the wire.
  c. Solder one end of the wire to the head of the screw.
    i. Place the screw on a table such that the shank is pointed upwards.
    ii. Solder the tip of the wire to the edge of the head (Figure 4A).

***Alternatives:*** If only one recording electrode is to be implanted, we recommend using a 3-pin female connector for the recording electrode (two pins for the electrode wires and one pin for the ground screw; Figure 4D). If multiple recording electrodes are to be implanted, we recommend soldering one ground screw to a connector with multiple pins (one for each electrode) rather than preparing multiple ground screws.

**Figure 5:**
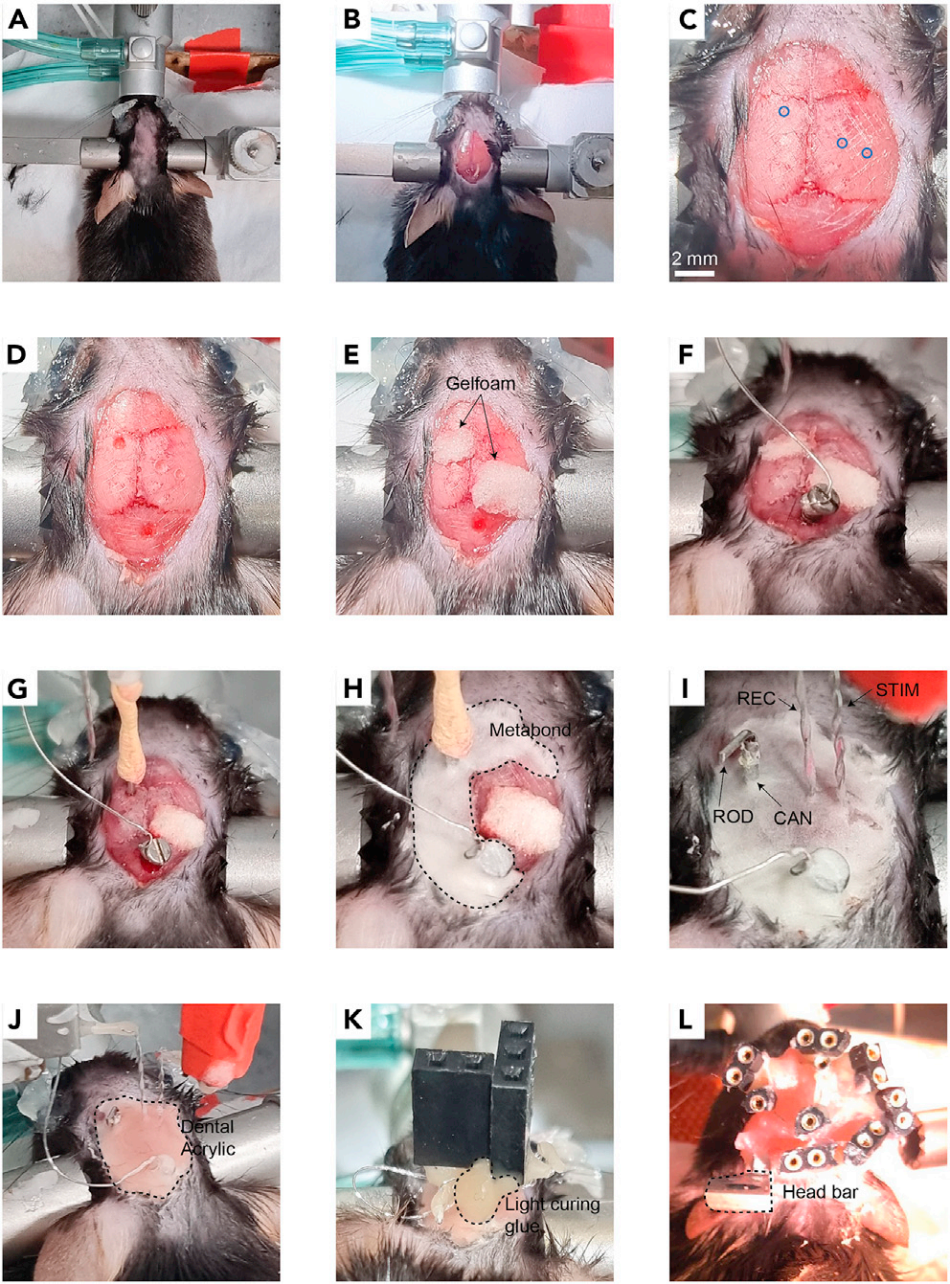
Surgical procedure to chronically implant a cannula and fEPSP electrodes in mice (A) Place the mouse in the stereotaxic instrument and remove the hair from the animal’s scalp. (B) Cut the scalp. (C) Expose the skull with hydrogen peroxide and perform incisions to the skull with a scalpel. Mark the implant coordinates with a pen (blue circles). (D) Drill holes in the skull for the implants. (E) Cover the holes with Gelfoam. (F) Insert the ground screw. (G) Insert the cannula. (H) Cover the cannula and ground screw with Metabond (dashed line). (I) Cover the electrodes with Metabond once they are localized at their final position. REC – recording electrode; STIM – stimulating electrode; CAN – cannula; ROD – filler rod. (J) Cover all implants with dental acrylic (dashed line) before releasing the electrodes from the manipulators. (K) Position the connectors at their final position close to the skull with light curing glue. (L) Finalize the head construct with dental acrylic. The photograph in this panel depicts a mouse with three pairs of electrodes for simultaneous recordings of evoked responses from three different pathways. Note the metal head bar (dashed line) used for connecting the mouse to the head fixation apparatus. This construct weighs approximately 2.3 gr, measured as the difference in the animal’s weight before and after the surgery. As a general guideline, head constructs should weigh no more than 10% of the total animal weight.
    **Figure 6:**
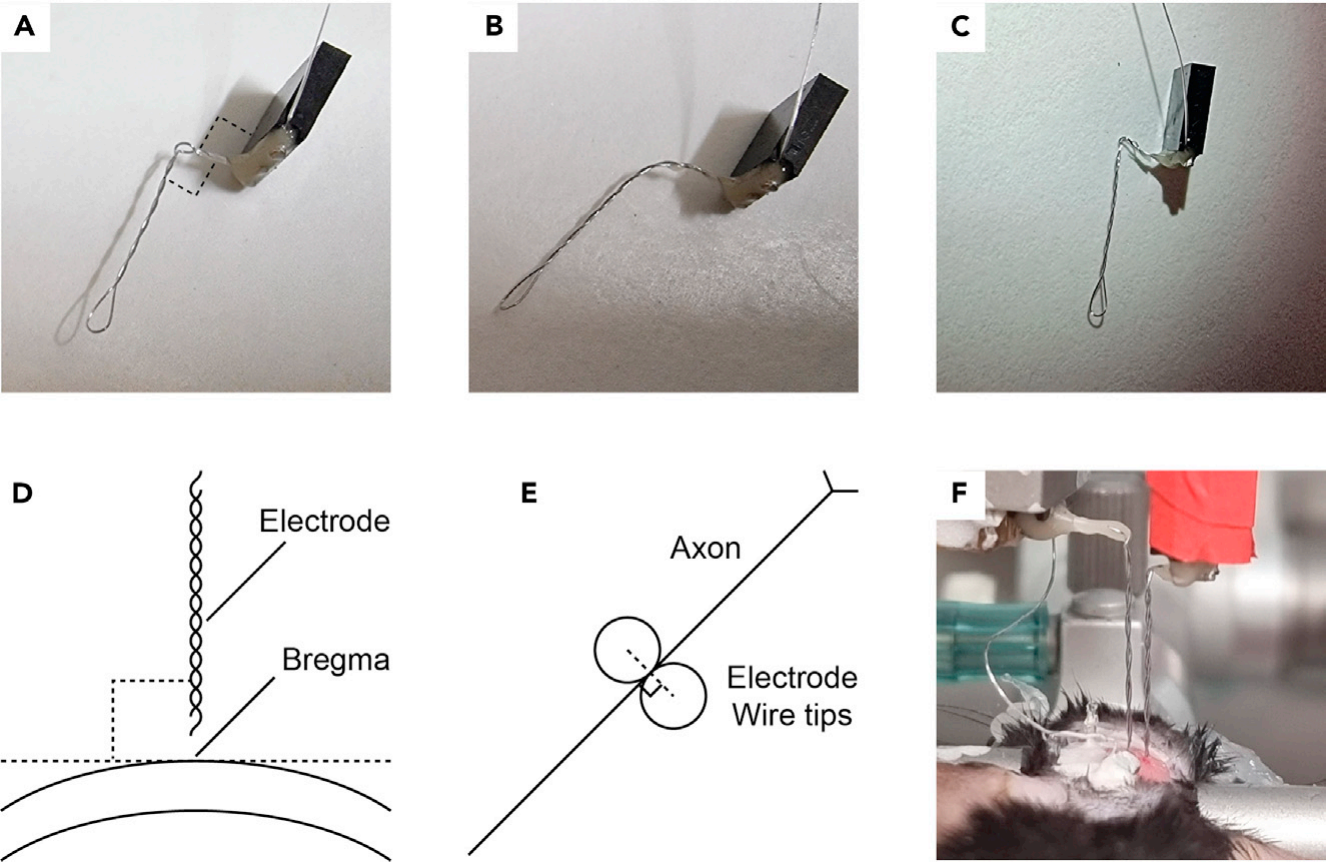
Alignment of electrodes during implantation (A) An electrode bent properly. Note two sharp right angles (dashed lines). (B) An electrode bent improperly with a round bend. This may cause the electrode to deviate from the DV axis when penetrating the brain. (C) An electrode bent improperly. Although the electrode tip is parallel to the connector, the deviation from 90° angles may cause the electrode to deviate during implantation. (D) Electrodes should be perpendicular to the bregma-lambda plane in both the AP and LM axes. (E) The line between the wire tips of the stimulating electrode should be perpendicular to the orientation of the axon bundle. If the orientation is unknown or disorganized, set the stimulating electrode such that the line between its wire tips is parallel to the interaural line. This default setting is arbitrary but may still help reduce variability between mice. (F) Alignment of electrodes during implantation should be done very patiently on all three axes.
  h. Cut the end of the wires according to the electrode purpose.
    i. For a recording electrode the cut should be 1 mm graded. This is achieved by cutting each wire individually (Figure 4F).
    ii. For a stimulating electrode the cut should be straight. This is achieved by cutting both wires with a single cut.
      ***Alternatives:*** The wires of recording electrodes are graded so that during implantation one wire is positioned at the target site and one wire is positioned at a distant site inert to the stimulation. Using the SC synapse as an example, the wires should be positioned at CA1 *stratum radiatum* and the sensory cortex 1 mm dorsal to CA1 (Figure 3C). Alternatively, the ground screw described in step 1 may provide the reference potential for both wires such that two fEPSP signals are generated from one implant. This is common practice when recording from regions other than the hippocampus. In the hippocampus, the use of an inert wire as the reference typically produces cleaner signals mainly because noise is manifested similarly on conductors with identical properties.
      ***Alternatives:*** To reduce tissue damage during implantation, or if the target brain region is very small (e.g. midline thalamic nuclei; Figure 7E) and/or very localized LFP events are of interest (e.g. hippocampal ripples), then a smaller diameter wire may be preferred (e.g. 790900 from A-M systems).
      ***Note:*** After an electrode is completed, measure resistance with a multimeter between the tip of each wire and the connector pins to make certain that conductance is exclusive to the appropriate pairs of wire and pin. For easy access to the connector and wire tips, connect a male connector to the female connector and attach rigid metal wires to the multimeter probes. This step cannot replace step 1 in the Prepare for surgery section.

2. Prepare electrodes.
  a. Cut 10 cm of stainless-steel wire.
  b. Expose 3 mm from both sides of the wire as in step 1.b.
  c. Loop the exposed ends of the wire as in step 1.d.
  d. Solder each end of the wire to adjacent pin connectors as in step 1.e. When bringing the second end of the wire in proximity to the second pin, make sure the wire is evenly bent throughout its length rather than folded at a single junction (Figure 4D).
  e. Twist the connector relative to the farthest end of the loop to a thread pitch of ∼0.5 (i.e., 1 complete turn every two mm).
    ***Note:*** Twisting the electrode can be done manually or with a magnetic stirrer (Figure 4E). The number of twists can be approximated as the length of the wire [mm]/thread pitch.
  f. Cover the pins and soldered wires with light curable glue as in step 1.f.
  g. Bend the electrodes to 90° twice: at 2- and 10-mm distance from the connector (Figure 6A). This is so that during implantation the electrodes can be spread apart from one another (Figure 5I). Use a table or other flat surface to assure right angles.
    **CRITICAL:** Inserting the electrodes at a 90° angle to the skull surface is crucial for reproducibility (see troubleshooting 5; Figure 6). This is mainly achieved by carefully aligning the electrodes to the manipulator during the surgical procedure (Figure 6F). Still, electrodes with round bends (Figure 6B) or bends other than 90° (Figure 6C) are more susceptible to deviate from the DV axis due to forces exerted by the brain during implantation.

3. Prepare a cannula and a filler rod.
  a. Cut ∼7 mm of the cannula tube (HTX-23T-30) with a cutter.
  b. File both ends of the tube with the rotary tool cutting wheel (Figure 4G).
  c. Polish both ends of the tube with a coarse grit sandpaper.
  d. Clean the interior side of the tube with water.
  e. Dry the tube completely with an air duster.
  f. Measure and log in a notebook the length of the cannula (Figure 4H).
  g. Prepare the filler rod from tube (HTX-28R-30) as in steps a-e.
  h. Bend the filler rod 2 mm from its edge to a ∼90° angle so that it can be placed within the cannula without falling deeper than intended (Figure 4I).

**Figure 7:**
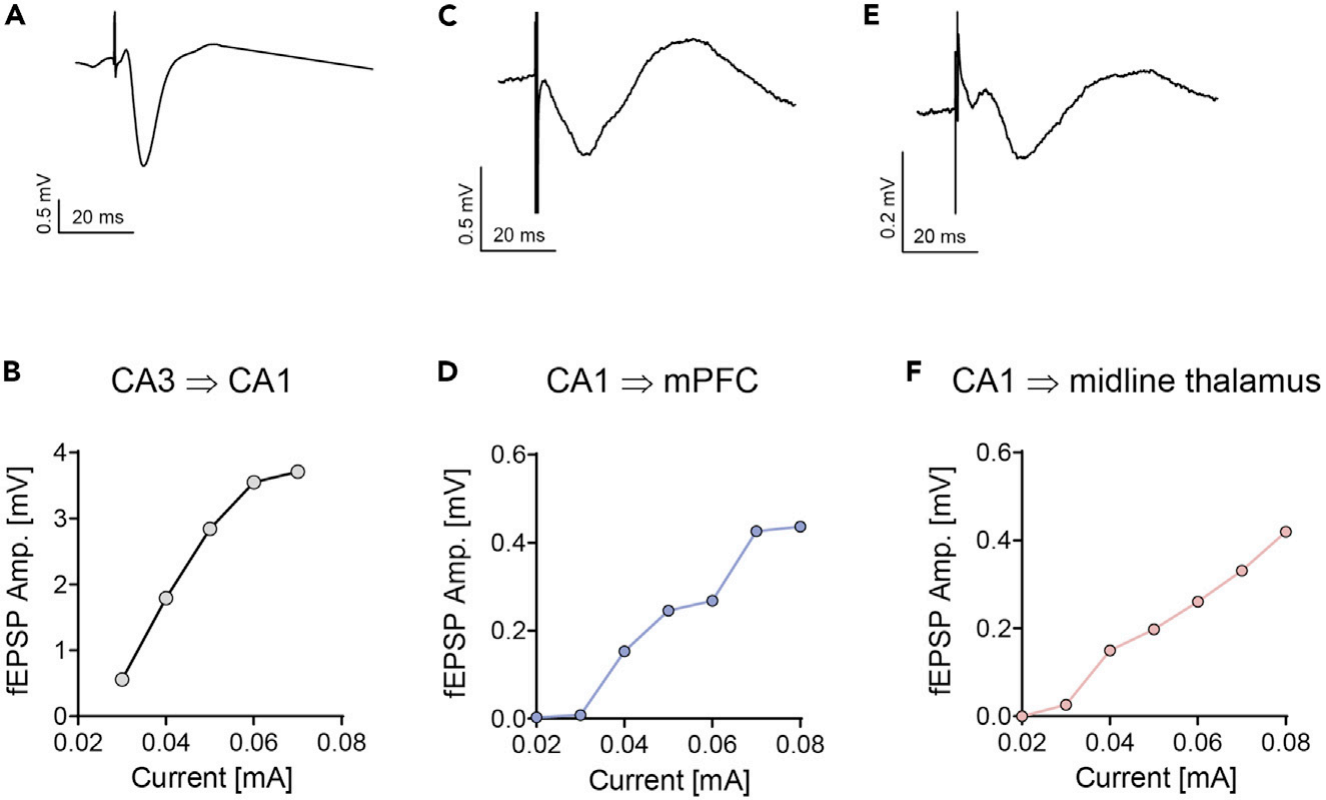
Example fEPSP recordings from three different pathways (A) An evoked response recorded from CA1 *stratum radiatum* in response to a 0.5 ms square pulse at 0.05 mA to the SC. (B) Evoked response amplitudes in response to increasing stimulating currents to the SC. This graph is termed an input-output (I/O) curve. (C) An evoked response recorded from the medial prefrontal cortex (mPFC) in response to a 0.5 ms square pulse at 0.07 mA to CA1 *stratum radiatum*. (D) I/O curve for the CA1 – mPFC pathway. (E) An evoked response recorded from midline thalamic nuclei in response to a 0.5 ms square pulse at 0.04 mA to CA1 *stratum radiatum*. (F) I/O curve for the CA1 – midline thalamus pathway.

### Surgery

**Timing: 2–6 h**

This step describes the implantation of fEPSP electrodes and cannulas. The surgery can be “chronic”, i.e., the animal is awakened and kept for future experiments, or “acute”, i.e., the experiment (e.g., injection of pharmaceuticals) is done during surgery while the animal is anesthetized.

4. Anesthetize the mouse.
  a. Place the mouse in a container connected to the isoflurane vaporizer and deliver 3% Isoflurane at a rate of 350 mL/min until the animal lies still in the container and the respiration rate becomes slow.
  b. Make sure that the animal is unresponsive by tipping the container.
  c. Place the mouse on the stereotaxic instrument.
  d. Set the heating pad to 34°C and insert the rectal probe.
  e. Inject i.p. the Ketamine/Xylazine solution.
  f. Gradually reduce the concentration and flow rate of Isoflurane to 1% at 150 mL/min.

**CRITICAL:** Throughout the procedure, monitor the animal’s anesthesia depth by occasionally testing the toe pinch reflex and observing its respiration rate.

5. Prepare the mouse for incision of the scalp (Figure 5A).
  a. Fixate the head of the mouse to the stereotaxic instrument.
  b. Inject s.c. the Carprofen solution.
  c. Spread eye protecting cream on the animal’s eyes.
  d. Remove all hair from the scalp with scissors and/or hair removal cream.
  e. Clean the surface of the scalp with povidone-iodine and wash with ethanol 70%.
6. Expose the skull.
  a. Make an incision along the midline of the scalp from the area between the eyes to the back of the head with a scalpel or scissors.
  b. Spread both sides of the scalp with bulldog serrefines.
  c. Remove the aponeurosis and periosteum with a drop of 3% hydrogen peroxide and clean with PBS. You may also use the blunt end of a scalpel to make sure all connective tissue has been removed (Figure 5B).
  d. Perform horizontal and vertical incisions to the skull with a scalpel without penetrating the skull. This strengthens the adherence of Metabond to the skull (Figure 5C).
7. Mark implant coordinates.
  a. Measure and log the anteroposterior (AP) distance between bregma and lambda. For adult (26–30 g) mice the distance should be 3.7–4.7 mm (Paxinos and Franklin, 2004).
  b. Align the head on all three axes.
    i. For pitch check that bregma and lambda are aligned in the dorsoventral (DV) axis.
    ii. For yaw check that bregma and lambda are aligned in the lateromedial (LM) axis.
    iii. For roll check that the DV height is identical ±2.5 mm lateral to bregma.
  c. Connect a needle to the manipulator and mark the desired coordinates for all implants (electrodes, cannula/s, and ground screw). Do this by applying ink to the needle tip (Figure 5C).

***Note:*** For convenience, we deposited in the accompanying GitHub repository a spreadsheet that can be used to align the skull and calculate distances relative to bregma (see resource availability).

8. Prepare craniotomies (Figure 5D).
  a. Hold the drill by hand or with a stereotaxic manipulator and slowly lower to the skull.
  b. Gradually remove bits of skull by alternately lowering and retreating the drill. This prevents overheating of the skull.
  c. Clean the skull with PBS.
  d. Cover the craniotomies with Gelfoam soaked in PBS (Figure 5E). This (1) coagulates the brain surface in case of bleeding, (2) maintains the brain tissue wet while the electrodes are mounted to the stereotaxic instrument, and (3) protects the craniotomies from the Metabond applied in step 11.

***Note:*** All implants described in this protocol fit best with a 0.7 mm diameter burr. Any modifications to the implants should also consider the burr size.

***Note:*** In mice, the dura is easily penetrated by both the electrodes and cannula. If the surgery is done on rats and/or the implants are modified to be less rigid (e.g. when using smaller diameter wires as electrodes), remove the dura with fine curved tweezers before step 8.d.

9. Insert the ground electrode to the hole with a screwdriver. Leave a small gap between the screw head and the skull (Figure 5F).
10. Implant the cannula to its proper location (Figure 5G).
  a. Connect a small needle (e.g., 27 gauge) to the manipulator.
  b. Pass the needle through the cannula until the tip of the needle almost emerges from the other side of the cannula. Glue the cannula in place with adhesive tape or plasticine.
  c. Insert the cannula into the brain until the proper coordinates are reached.

**CRITICAL:** Lowering an implant such as a cannula or electrodes into the brain must occur very slowly in order to minimize brain damage and dimpling; two major contributors to intra- and inter-subject variability (see troubleshooting 4 and 5, respectively). As a rule of thumb, we recommend advancing 300 um every 10 s and even slower as the target depth is approached.

**CRITICAL:** Validate the length of the cannula before implantation. This measurement will be used during the experiment to direct the syringe needle precisely through the cannula (step 20.b).

11. Secure the cannula and ground screw with Metabond (Figure 5H). Be careful not to cover any other craniotomies (e.g., for the electrodes). If Metabond reaches other craniotomies, remove the Gelfoam from the craniotomies before the Metabond cures and cover the craniotomies with a small drop of PBS.

**CRITICAL:** For maximal adherence to occur, make sure that the skull is completely dry before applying Metabond.

***Note:*** Most stereotaxic instruments allow the control of two manipulators. Thus, only two devices can be implanted simultaneously since they must be secured with Metabond to the skull before they are released from the stereotaxic manipulator. If multiple electrodes and/or cannulas are to be implanted, plan in advance the order of implantations such that Metabond is applied as few times as possible. For example, if only two electrodes and a ground screw are implanted, Metabond can be applied only once after positioning of the electrodes (step 14).

***Note:*** Most brain atlases provide DV coordinates starting from the skull surface at bregma. From our experience, calibrating depth coordinates from the brain (pia) surface reduces variability, especially for brain regions positioned laterally.

**CRITICAL:** We highly recommend performing histological verification of the implants’ position after sacrificing the animal (troubleshooting 5; Figure 2). To enhance the detection of the electrodes in a histological slice, dip the electrodes in a cell labeling solution (e.g. Vybrant CM-Dil by Invitrogen) prior to implantation (step 12).

**Pause Point:** Take a break for 15–20 min while the Metabond is being cured. Do not be tempted to touch the implants even if the outer surface of the Metabond appears dry (see troubleshooting 5).

12. Insert the electrodes to the brain.
  a. Connect the electrodes to the electrophysiological setup. Make sure the cables are long enough to permit free movement of the electrodes.
  b. Mount the recording and stimulating electrodes on manipulators.
  c. Move the electrodes towards their holes and manually adjust their alignment.
    i. Both electrodes are perpendicular to the bregma-lambda plane in both the AP and LM axes (Figure 6D).
    ii. The line between the wire tips of the stimulating electrode is perpendicular to the direction of axon travel (Figure 6E).
    iii. The recording and stimulating electrodes are spaciously positioned such that they will not collide during implantation (Figure 6F).
  d. Insert the electrodes 200–300 um into the brain.

**CRITICAL:** If the target region involves midline structures (e.g. thalamic nuclei), the implants must be inserted in an angle to prevent puncturing of the sagittal sinus. This should be done by adjusting the angle of the manipulator. Do not bend the electrodes to the desired angle as this will not hold true during implantation.

13. Search for an evoked response.
  a. Lower the stimulating electrode 0.5 mm above the target depth.
  b. Start recording while delivering a medium (0.04–0.1 mA) intensity stimulus at 0.5 Hz.
  c. Lower the recording electrode to 0.5 mm above the target depth.
  d. Lower the stimulating and recording electrode one at a time in graduations of 0.05 mm until the “best” signal is achieved (see CRITICAL remark below and troubleshooting 1).

**CRITICAL:** The evoked response of commonly investigated pathways (e.g. the SC) frequently exhibit characteristic waveforms which can be used to determine that the electrodes are in their proper position (Figure 7A). Specifically, four main parameters should be inspected while searching for a signal (step 13): (1) the delay of the response relative to stimulus onset, (2) the presence of STP (facilitation versus depression, depending on the frequency of stimuli and the type of synapse under investigation), (3) the waveform shape, and (4) the amplitude. For novel pathways, the main indication that the target has been reached is the evoked response amplitude. However, since a large response can be achieved by accidently recording other pathways, we recommend not to deviate from the DV coordinates by more than 500 um even if it produces larger response amplitudes.

**Figure 8:**
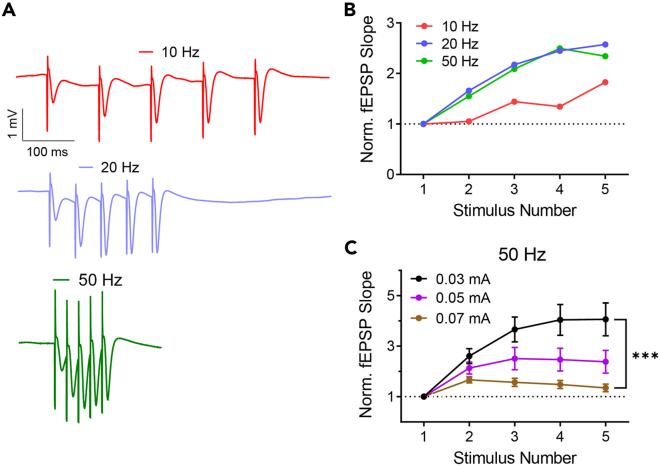
Representative fEPSP recordings for investigating short-term plasticity and its modulation by stimulus frequency and intensity (A) Traces of fEPSP recordings in response to a train of five stimuli delivered at various frequencies. Red – 10 Hz; blue – 20 Hz; green – 50 Hz). Each trace is the average of 10 repetitions delivered with a 30 s inter-burst interval. (B) Quantification of short-term synaptic plasticity (STP) is typically done by measuring the slope of each response and normalizing it to the slope of the first response. Synaptic facilitation is defined as a ratio greater than one and synaptic depression is defined as a ratio smaller than one. In the SC synapse, a train of stimuli at 20 Hz and 50 Hz typically elicits synaptic facilitation to a greater extent than a train of stimuli at 10 Hz. (C) Quantification of synaptic facilitation in response to various stimulus intensities (50 Hz burst). Black – 0.03 mA; purple – 0.05 mA; Brown – 0.07 mA. Error bars represent SEM.

***Note:*** All pathways investigated in our lab displayed some degree of synaptic facilitation when an STP protocol was applied (Figure 8A). If synaptic facilitation is to be expected, STP protocols during implantations may serve as another indication that the target regions have been reached.

14. Once the desired signal is achieved, check stability of the signal by delivering a stimulus every 20 s for 5–10 min.

**Pause Point:** Take a break for 5–10 min.

**Figure 9:**
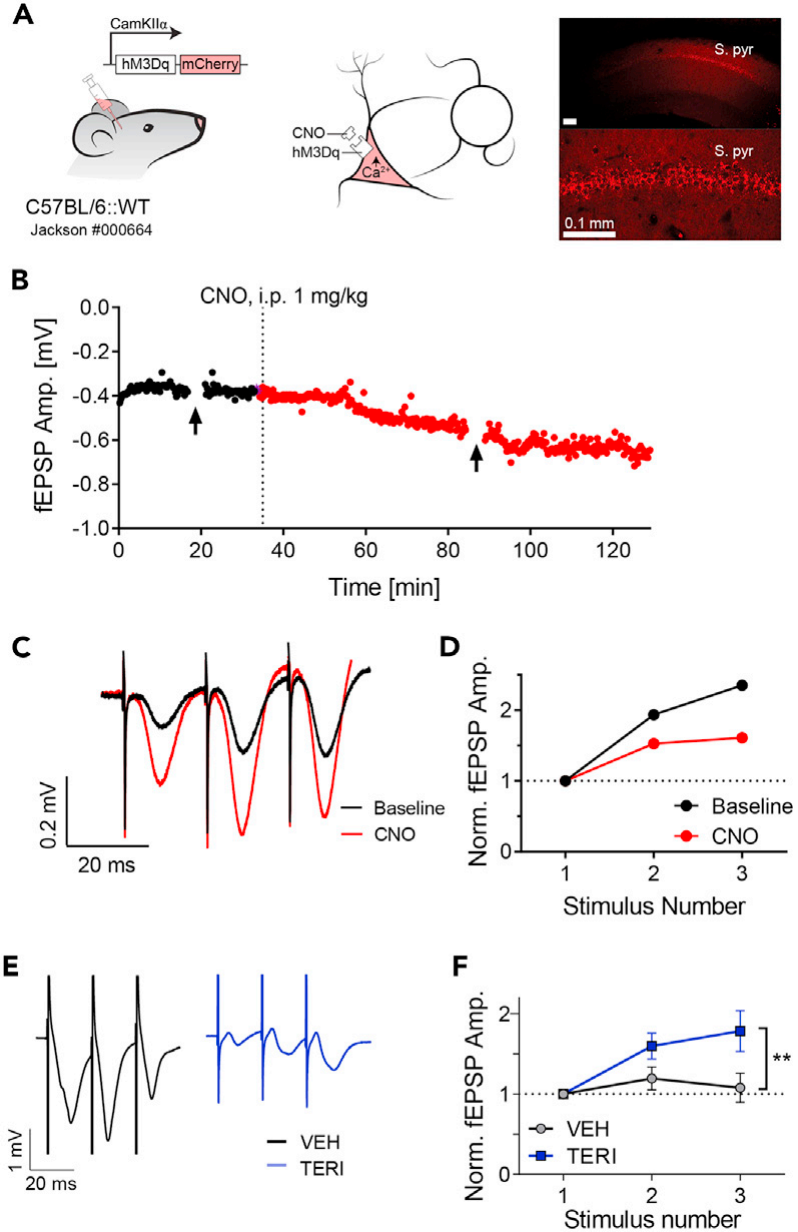
fEPSP recordings of the SC in response to pharmacogenetic/pharmacological manipulation (A) Pharmacogenetic manipulation of CA1 pyramidal cells. Left: mice were injected with AAV5-CamKIIα-hMD3q-mCherry in CA1 to express hMD3q specifically in pyramidal cells. Middle: in the presence of the ligand CNO, hMD3q is activated and leads to an increase in intracellular calcium. Right: Histological image at ×10 (top) and ×40 (bottom) magnification of CA1 from a mouse expressing the chemogenetic agent. (B) fEPSP amplitudes recorded at the SC synapse in response to a 0.5 ms square pulse delivered once every 15 s. Dashed line represents the time of i.p. injection of 1 mg/kg CNO. Arrows represent the time of measurements for the data in parts (C and D). Note that continuously monitoring evoked responses can reveal the kinetics of a manipulation. In this example, CNO started to elicit a noticeable effect approximately 15–20 min after the i.p. injection. (C) Representative fEPSP traces in response to a train of three stimuli delivered at 50 Hz before (black) and after (red) CNO administration. (D) Synaptic facilitation before (black) and after (red) CNO administration. CNO decreased the amount of synaptic facilitation despite increasing the absolute amplitude of the first response. (E and F) Teriflunomide (TERI) or the same volume of vehicle (VEH) was injected intracerebroventricular daily for 3 consecutive days. fEPSP recordings were done 2–4 h after the last injection. Adapted from (Styr et al., 2019). (E) Representative fEPSP traces in response to a train of three stimuli delivered at 50 Hz after TERI (blue) or VEH (black) administration. (F) Synaptic facilitation before (black) and after (blue) TERI administration. TERI increased the amount of synaptic facilitation despite decreasing the absolute amplitude of the first response. N = 9 mice for each group. Error bars represent SEM.

***Note:*** If an acute experiment is preferred, continue with the stimulation for 30 min or until a stable signal is achieved (i.e. the evoked response amplitude remains constant for at least 5 min; Figure 9B). Afterwards, continue with the experiment according to the guidelines detailed in the experiment section. Note that acute surgeries typically take > 5 hours and thus require that the animal be sacrificed at the end of the experiment.

15. Cover the electrodes and entire skull with Metabond (Figure 5I). All notes provided for step 11 are also relevant for this step.
16. Cover the electrodes and entire skull with dental acrylic (Figure 5J). Wait 10–30 min for the acrylic to be completely dry.
17. Finalize the head construct.
  a. Gently release the electrodes from the cables and manipulators.
  b. Position the connectors at their final position close to the skull and hold them in place with a few drops of light curing glue (Figure 5K).
  c. Secure all components including the head bar to one another and to the Metabond surface with dental acrylic (Figure 5L).

***Note:*** Use as little of dental acrylic as possible to minimize the weight of the final head construct, but enough dental acrylic to cover all wires.

18. Finalize the surgery.
  a. Apply povidone-iodine to the scalp at the edges of the head construct.
  b. Inject i.p. the buprenorphine solution.
  c. Set the Isoflurane concentration to 0%. Do not stop the airflow from the vaporizer.
  d. Wait for 5–20 min until the toe pinch reflex can be elicited.
  e. Weigh the mouse and log its weight in a dedicated notebook.
  f. Reposition the mouse on the heating pad until it is completely awake and moving.
  g. Place the mouse in its home cage.

**CRITICAL:** The added weight during surgery should be no more than 10% of the animal’s body weight.

**CRITICAL:** We typically allow the mouse to recover for 5–7 days before handling it. During this time the surgeon should check the animal’s wellbeing twice a day and log its recovery.

### Experiment

**Timing: 1–4 h daily, for 7 days up to 6 months**

This major step describes the conduction of experiments in mice implanted with fEPSP electrodes and a cannula. Here we focus on studying pharmacological agents but the general procedure can be easily expanded to a wide variety of other manipulations and/or behavioral settings. Before attempting any manipulations (step 20), we insist that the experimenter achieve stable baseline recordings for several consecutive days (step 19). Although the data acquired during this time is typically not reported, a stable baseline provides invaluable confidence when later trying to interpret the experimental results (See troubleshooting 4).

19. Perform baseline recordings.
  a. Connect the mouse to the electrophysiological setup.
  b. Place the mouse in the experimental arena (open field, home cage, etc.)
  c. Characterize the I/O curve (see the before you begin section).
  d. Analyze the I/O curve with the MATLAB analysis package provided in this study.
  e. Based on the I/O curve, select 1–3 stimulus intensities for STP recordings.

***Note:*** The head fixation apparatus can be used during recordings to minimize movement artifacts (troubleshooting 3) and variability in arousal state (troubleshooting 4). However, if the head fixation apparatus is to be used during recordings, the animal must be accustomed to the apparatus several days before starting the recording sessions.

***Note:*** The overall number of stimulations per day should be limited to avoid long term plasticity effects (troubleshooting 4). Thus, to minimize the amount of stimulations while retaining enough repetitions for statistical analysis, we recommend fully characterizing the I/O curve only 2–3 times a week (e.g. at the beginning of the baseline period, one day before the perturbation and immediately after the perturbation). Daily measurements should include only 3–5 stimulus intensities selected as representatives of the full I/O curve.

20. Perform the experiment.
  a. Perform a recording session as in step 19.
  b. Apply a sham (e.g., saline injection) or true manipulation (e.g., drug injection; Figures 9E and 1B).
    i. Prepare the syringe and drug delivery system with the desired solution (see the before you begin session).
    ii. Position the head fixation apparatus near the stereotaxic instrument and connect the animal.
    iii. Using the stereotaxic manipulator, direct the syringe towards the cannula opening.
    iv. Slowly lower the syringe to a depth precisely equal to the cannula length.
    v. Inject the solution and log in a notebook the volume, delivery rate, and time of injection.
    vi. Wait 10–20 min after the injection has been completed and before the needle is removed. This allows sufficient time for the drug to diffuse and minimizes the risk that pulling the needle will cause back propagation of the drug (troubleshooting 6).
    vii. Slowly raise the needle until it exits the cannula.
    viii. Release the animal back to its home cage.
  c. Wait as much time as needed for the drug to produce its expected effect (troubleshooting 6) and perform another recording session.

**CRITICAL:** Before and after every injection with an electrical pump, we recommend releasing a small volume (100–500 nL) of drug outside of the brain to make sure the needle is not clogged (troubleshooting 6).

**Figure 10:**
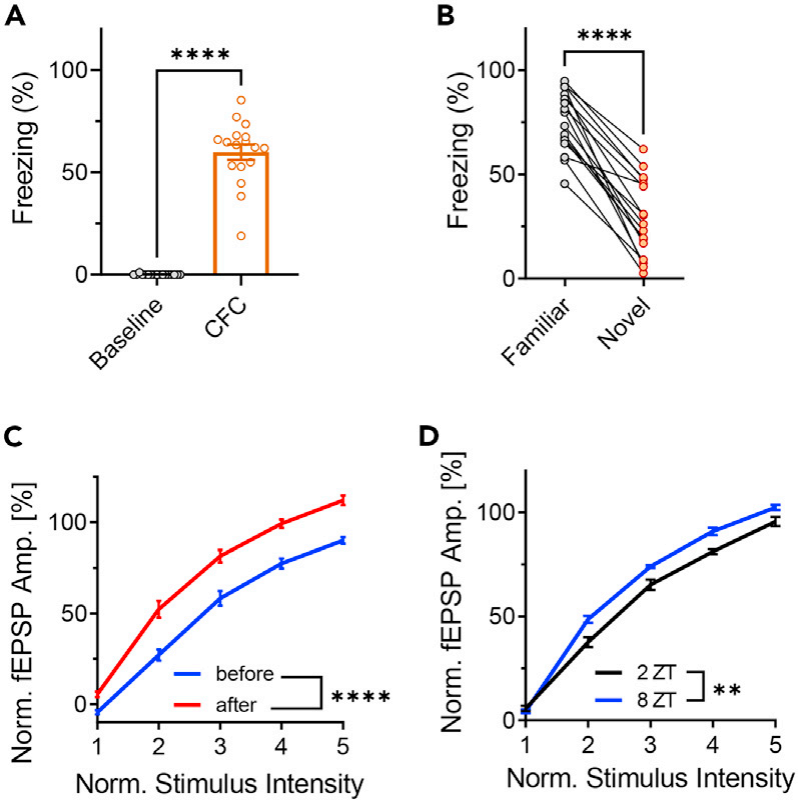
fEPSP recordings of the SC in response to behavioral interventions. (A–C) Contextual fear conditioning (CFC) increased synaptic transmission of the SC. In sum, mice were individually placed in an arena three times in two consecutive days. On the first day, after two min in the arena the mice received two 1 mA electric shocks 80 s apart through a metal grid placed on the floor. This typically causes the animal to associate the context (visual and olfactory cues of the arena) with an aversive stimulus (the electric shock). During the second day, mice were placed in the arena with the same contextual cues but no electric shock was delivered. Four hours later, the visual and olfactory cues were altered to represent a novel context and the mice were placed again in the arena for 2.5 min without an electric shock. The percent of time spent without movement (freezing) serves as a measurement for the association of the context with the electric shock (Curzon et al., 2009). fEPSP measurements were taken from head-fixed animals three times a day, two hr before and after the onset of behavioral experiments. Data presented is from 15 mice. (A) Percent freezing after CFC (orange) was significantly greater than before CFC (gray) in the same context (comparison of the behavioral session on day 1 with the first behavioral session on day 2). (B) Percent freezing in the context of the electrical shock (gray) was significantly greater than in a novel context (orange; comparison of the two behavioral sessions on day 2). (C) The I/O curve of the SC after CFC (red) was significantly greater than before CFC (blue). For each mouse, the average I/O curve from all sessions was used to normalize the I/O curve of a single session. For each session, the I/O curve was generated by five stimulus currents linearly spaced between values that elicit a minimal and maximal response. A significant difference in the evoked response amplitudes between the two recording times was found by two-way ANOVA with repeated measures (p < 0.0001). This is in accordance with previous findings (Subramaniyan et al., 2021) and implies that fear conditioning induced LTP of the SC. Error bars represent SEM. (D) The I/O curve of the SC from 13 mice was significantly greater eight (8 ZT; blue) compared to two (2 ZT; black) hours into the light phase. I/O curves were generated and normalized as in (C). This effect of the circadian rhythm on neural excitability is in accordance with previous findings (Herzog, 2007). Error bars represent SEM.

***Alternatives:*** If long term effects of the manipulation are of interest, we recommend performing a recording session once a day at the same time of day and comparing the data between days rather than comparing the data between two recording sessions of the same day. This increases statistical power since it allows for several days to be averaged and it also prevents differences between the first and second sessions that are due to the circadian rhythm (Figure 10D).

## Expected outcomes

In our lab, novel experimenters with no previous knowledge in electrophysiology typically master the technique of fEPSP recordings within 2–4 months of continuous training from a veteran student. Once the technique is mastered, approximately 80% of implanted mice will display a clean and stable signal for more than one month. The maximum time we recorded from a mouse with fEPSP electrodes was six months. We have not tested the protocol for duration beyond six months.

Multiple signals and experiments are easily accessible once this protocol is successfully implemented. Figure 7 demonstrates three pathways that can be targeted by altering the implantation coordinates: CA3 to CA1 via the SC (Figures 7A and 7B), CA1 to the medial prefrontal cortex (mPFC; Figures 7C and 7D), and CA1 to midline thalamic nuclei (Figures 7E and 7F).

Figure 8 demonstrates the ability to investigate short-term plasticity in the SC pathway by altering the stimulation protocol. Note that the degree of synaptic facilitation and/or depression can be modulated by altering the stimulus frequency and intensity.

Figure 9 demonstrates the effect of pharmacogenetic and pharmacological interventions on synaptic transmission of the SC. Figures 9A–9D depict the effect of administering Clozapine N-oxide (CNO) to a mouse injected with CamKIIa-hMD3q to area CA1. Figures 9E–9F depict the effect of Teriflunomide on synaptic transmission (Styr et al., 2019). Note that the pharmacokinetics of a drug can be investigated by continuously probing evoked responses (Figure 9B).

Figure 10 demonstrates the effects of behavioral interventions on synaptic transmission of the SC. Figures 10A–10C depict the effect of contextual fear conditioning (CFC) and Figure 10D depict the effect of the circadian rhythm.

**Figure 11:**
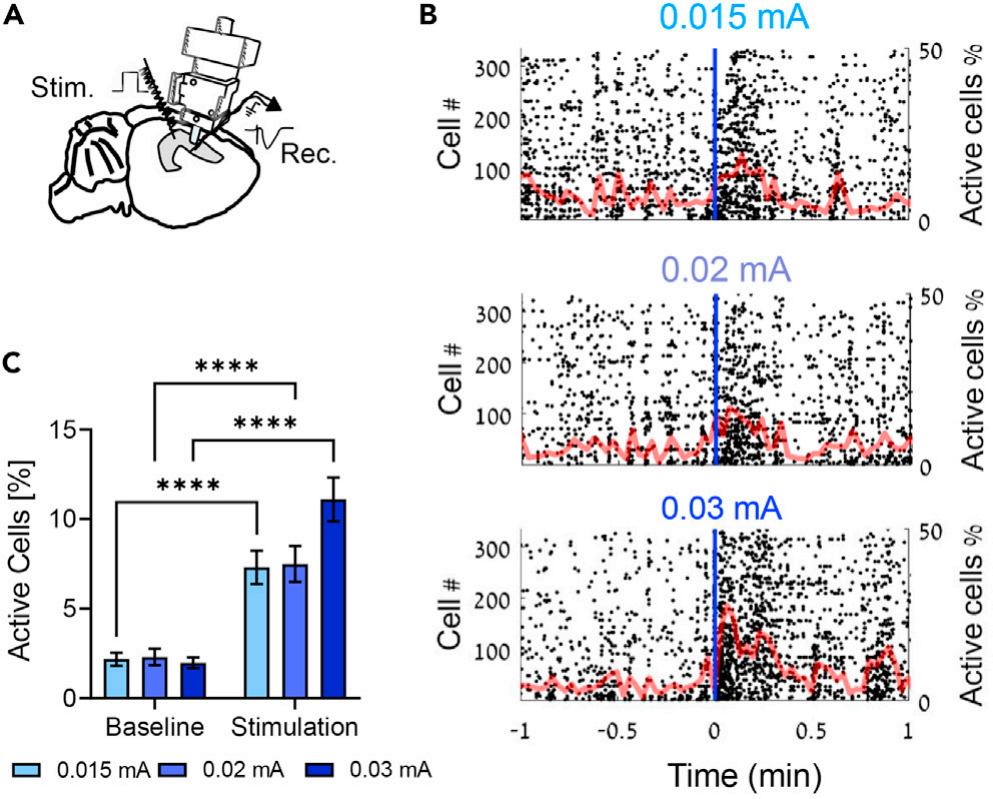
Stimulation of the SC pathway combined with CA1 calcium microendoscopy in behaving mice (A) Graphical diagram of a mouse brain implanted with fEPSP electrodes at the SC synapse and a head-mounted miniaturized fluorescence microscope at the ipsilateral CA1 *stratum pyramidale*. Prior to implantation the mouse was injected with AAV5-CaMKIIα-GCaMP6f to express the Ca^2+^ sensor GCaMP6f (Chen et al., 2013) in CA1 pyramidal neurons. (B) Representative raster plots of calcium transient activity. Dashed line represents the timing of current injection to the SC. Superimposed in red is the percent of active cells in each time bin (10 Hz temporal resolution). (C) The percentage of active cells significantly increased following stimulation of the SC for all stimulus intensities. Two-way ANOVA revealed a significant interaction with stimulation (p < 0.05). Error bars represent SEM.

Figure 11 demonstrates the ability to combine this protocol for fEPSP recordings with other modalities to record single unit activity, such as calcium imaging.

## Quantification and statistical analysis

Synaptic transmission strength is typically quantified as the amplitude (voltage difference between baseline and peak) and/or slope (e.g., between 20% and 90% of the peak) of the evoked response waveform. Although these parameters can be easily obtained from any given waveform, the voltage trace itself may contain other entities in addition to the evoked response, such as a stimulus artifact, fiber volley, and/or spontaneous activity. Further, the precise timing and shape of the evoked response frequently varies between mice and pathways. Accordingly, the analysis of fEPSP recordings is typically done by inspecting the raw traces and manually marking the points of interest. To facilitate this process without compromise, we designed and realized a MATLAB based package dedicated to fEPSP signals, including a graphical user interface for manually marking the baseline and peak of evoked responses. The analysis pipeline consists of four main steps:

1. Organize the raw data according to the number of recording channels and stimulus intensities.
2. Manually mark the start and peak of each evoked response.
3. Calculate the slope and amplitude of each trace.
4. Visualize the results.

To compare various mice or experimental groups, results stored during the analysis can be processed further in MATLAB or be easily transferred to other data analysis and visualization software.

Unless otherwise stated, statistical analysis throughout this paper was done by two-way ANOVA with Sidak’s multiple-comparisons test. ∗p < 0.05, ∗∗p < 0.01, ∗∗∗p < 0.001, and ∗∗∗∗p < 0.0001; ns, non-significant (p > 0.05). Error bars represent SEM.

## Limitations

As previously discussed, one of the main advantages of this protocol is the simplicity and versatility of our custom electrodes which are composed of relatively thick (76–127 um) wires and thus can be implanted directly at the target without the need for specialized microdrives. However, wire diameter also determines the volume of brain tissue that is stimulated and summed to produce the fEPSP signal (Buzsáki et al., 2012). This characteristic of low spatial resolution, combined with the non-specific nature of electrical stimulation, may produce an evoked response that includes activity from regions other than those intended. Consequently, if the desired manipulation (e.g., candidate drug) produces a subtle effect on synaptic transmission, or if the effect is specific to a small sub-region, it may not be detectable in the fEPSP signal. Thus, we suggest researchers to consider alternative explanations when interpreting negative results since in some instances this protocol may exhibit a suboptimal true negative rate.

## Troubleshooting

### Problem 1

The signal is contaminated by magnetic interferences (step 19).

### Potential solution

The quality of electrophysiological recordings is determined, above all, by the signal-to-noise ratio (SNR). Amongst the major contributors to noise are those attributed to electromagnetic interferences, specifically the power line interference (PLI; Rangayyan, 2009). At the analysis stage, PLI can be treated by removing oscillatory interferences in the frequency domain (e.g., via notch filters; Widmann et al., 2015) or in the time domain (e.g., via time synchronous averaging; Tal and Abeles, 2013). Preferably, at the acquisition stage PLI can be prevented by “shielding” with a Faraday cage the analogue signal from its point of origin up until it is digitized (Chimene and Pallas-Areny, 2000; Huhta and Webster, 1973).

Importantly, most electrophysiological setups require that all shields share precisely the same ground. This includes, for example, the copper strands in data cables, the copper mesh surrounding a behavioral arena, the amplifier chassis, etc. Otherwise, even small potential differences between shield references may result in unwanted current (referred to as a “ground loop”) and be detrimental to the root mean square (RMS) of the signal. Thus, before starting an experiment, and especially if the electrophysiological setup is new or modified, we highly recommend dedicating as much time as necessary to assure all relevant components are securely connected to a common ground.

For each component of the electrophysiological setup, consult with the manufacturer and/or other researchers if the component should be connected to the same reference as the animal and if that reference should be connected to earth.

Record a signal from electrodes immersed in PBS and continuously observe the raw signal in a time scale of 1 s while performing step 3.

Start connecting the shields of all components as decided in step 1. From our experience, a 26 AWG wire (e.g., 2843/19 by Alpha Wire) is sufficient for shortening references. Typically, a noticeable effect on signal quality will be observed only after all components are properly grounded.

Record ∼5 min of raw data and compute the signal’s power spectrum and RMS. Save the results for future comparisons. Clean signals should exhibit an RMS of 0.05–0.15 mV, but higher values up to 0.3 mV are also acceptable for synapses that elicit high amplitude evoked responses (e.g., the SC; Figure 7).

### Problem 2

No fEPSP signal is found during surgery (step 13).

### Potential solution

If no fEPSP is detected when the electrodes reach their final depth, do not continue to search for a signal but rather leave the electrodes at their precise coordinates without delivering current for 15–30 min. This provides an opportunity for the neural tissue to recover from acute dimpling or trauma caused by the initial implantation. Afterwards, resume step 13 (deliver a 0.5 ms square pulse @ 0.5 Hz) and gradually increase the stimulus current until a signal is detected or the current level reaches 0.03 mA.

If still no fEPSP is detected, lower the stimulating electrode in graduations of 50 um to a depth no greater than 300 um or 30% of the target depth.

If still no fEPSP is detected, slowly raise the stimulating electrode until a signal is found or the electrode is removed from the brain. Then, replace the stimulating electrode with a new one and gradually lower it to its final depth as described in the protocol.

If still no fEPSP is detected, repeat step 3 for the recording electrode; slowly raise the recording electrode until a signal is found or the electrode is removed from the brain. Then, replace the recording electrode with a new one and gradually lower it to its final depth as described in the protocol.

***Note:*** In some recording setups, electromagnetic interferences are amplified when the recording electrode is suspended in air rather than immersed in solution (PBS or brain tissue). If this is expected but does not occur when the electrodes are removed from the brain, it implies that the absent signal is due to a technical problem with the electrode and/or recording setup.

If still no fEPSP is detected, remove both electrodes and re-examine the skull alignment and coordinates of the craniotomy. If no error is detected, you may try to repeat steps 3 and 4 though it is highly unlikely that two electrodes prepared in the same batch should fail and the third succeed. Alternatively, inspect the electrophysiological signal as described in the before you begin section and/or by connecting a mouse with an established signal.

If the pathway under investigation is a relatively new target, inject 500 uL of ink or dye (e.g., Vybrant CM-Dil by Invitrogen) at the coordinates of the electrodes and sacrifice the animal for the purpose of histological examination.

### Problem 3

Motion artifacts contaminate recordings in active awake mice (step 19).

### Potential solution

Motion artifacts typically manifest as rapid, variable deflections and as such they are extremely difficult to remove at the analysis stage. At the acquisition stage, motion artifacts originate from multiple sources (Nicolai et al., 2018) some of which are inherent to the selected methodology (e.g., the interface between the electrodes and brain parenchyma). Using this protocol, motion artifacts are typically negligible relative to the evoked response and thus should not be a major concern. However, motion artifacts may become more severe when a ground screw is used as the reference potential instead of an electrode wire (see Alternatives note to step 2.h). Still, from our experience, motion artifacts can be diminished significantly by meticulously minimizing the degrees of freedom between all moving parts, specifically between the implants and the skull and between the electrode connectors and their associated cables.

### Problem 4

fEPSP signals are variable across days (step 19).

### Potential solution

The major contributors to intra-subject variability are inflammation, synaptic plasticity, behavioral state, and electrophysiological instrumentation.

Inflammation drastically affects tissue excitability (Galic et al., 2012; Isbrandt, 2017). Thus, even animals that appear to have recovered completely from surgery may still suffer from neuroinflammation. Wait an additional week or two before attempting to achieve a stable baseline.

Identify and perform daily tests on electrophysiological components that are prone to instability. Two immediate suspects should be all battery powered components (e.g., stimulator, amplifier, etc.) and cables with loose connections.

To assure synaptic plasticity does not occur during a recording session, reduce the overall number of stimulations provided in a session and re-examine the stability between recording sessions.

Excitability in the mammalian nervous system is frequently modulated by the behavioral state of an animal (Dash et al., 2018; Subramaniyan et al., 2021) and/or its circadian rhythm (Herzog, 2007).

Transfer the animal to the experimental room at least 30 min before starting a recording session.

Be strict concerning the zeitgeber time of a daily recording session (Figure 10D).

Assure that the animal is accustomed to the experimenter, experimental room, and electrophysiological setup. This is typically done by connecting the animal to the electrophysiological setup for 30 min every day for seven days without providing any stimuli.

Log in a notebook any unique interference, such as ambient noise or unexpected entrances to the experimental room.

In general, we highly recommend running experiments on multiple mice simultaneously while counterbalancing the order of recordings within each day. This is extremely helpful at revealing intra-subject variability due to methodological errors since these typically manifest similarly in all subjects.

### Problem 5

fEPSP signals are variable across animal subjects (step 20).

### Potential solution

Inter-subject variability typically occurs due to the electrodes or, more commonly, the electrodes’ position in the brain.

When constructing the electrodes take specific care of step 2.h. The height difference between wires should be managed with minimal tolerance. In addition, cutting the wires should be done with very sharp scissors and at a right angle so that the surface area of their tips is invariable.

When implanting the electrodes, take specific care of step 7 (aligning the skull), step 12 (aligning the electrodes) and step 15; do not touch anything near the electrodes until the Metabond has been cured.

Histological verification of the implants’ position must be done on all animal subjects. The implants of this protocol can be dipped in a cell labeling solution (e.g., Vybrant CM-Dil by Invitrogen) prior to implantation to enhance their detection in a histological slice (Figures 3C and 3E), though this is typically not necessary as their signature is readily noticeable even with standard light microscope under ×10 magnification.

### Problem 6

The manipulation does not exhibit an effect on synaptic transmission (step 20).

### Potential solution

The recording sessions described in step 19 are given as a snapshot in time. Thus, they should be carried out in accordance with the pharmacokinetics of the drug and/or any other factor that can help predict the timing of the desired effect. If the kinetics are unknown or are suspected to be other than that predicted, a preliminary experiment can be conducted such that a stimulus is given every 20 s for 1–6 h before and after the manipulation is applied (Figure 9B).

Positive controls, such as administering a drug with a known effect on excitability, are extremely useful in revealing methodological errors as the cause for negative results. For example, in our lab administrating DNQX always induces a detectable decrease in synaptic transmission due to the blockade of AMPA receptors.

Lastly, as discussed in the Limitations section, negative results could be attributed to the low specificity of fEPSP recordings. However, mastering this protocol and specifically the surgical technique can significantly decrease the chance to encounter false negatives. First, non-specific excitation generated during electrical stimulation of the brain could mask the desired effect. Accordingly, precise localization of the electrodes during surgery reduces the absolute amount of current necessary to generate an evoked response and thus minimizes the extent of non-specific excitation. Indeed, our experience shows that novice surgeons frequently produce signals comparable to veteran surgeons only when applying higher stimulus intensities. Second, averaging data from multiple subjects can reveal effects that are otherwise too small to be revealed by any individual mouse. For a given effect size, accurately reproducing experimental conditions and signal parameters (troubleshooting 3, 4 and 5), as naturally occurs with time and practice, reduces the proportion of unexplained variance and thus the number of mice (i.e., sample size) necessary for an effect to emerge.

## Resource availability

### Lead contact

Further information and requests for resources and reagents should be directed to and will be fulfilled by the lead contact, Inna Slutsky (islutsky@tauex.tau.ac.il).

### Materials availability

The CAD files, stim protocols, and coordinates spreadsheet used in this paper are available at: https://github.com/leoreh/slutsky_fepsp.

A version of record of the repository at the time of publication can be found at: https://zenodo.org/badge/latestdoi/414165259.

## Data Availability

The code used to analyze the results described in this manuscript is available at: https://github.com/leoreh/slutsky_fepsp. A version of record of the repository at the time of publication can be found at: https://zenodo.org/badge/latestdoi/414165259.
